# Extracting microRNA-gene relations from biomedical literature using distant supervision

**DOI:** 10.1371/journal.pone.0171929

**Published:** 2017-03-06

**Authors:** Andre Lamurias, Luka A. Clarke, Francisco M. Couto

**Affiliations:** 1 LaSIGE, Faculdade de Ciências, Universidade de Lisboa, Lisboa, Portugal; 2 BioISI: Biosystems & Integrative Sciences Institute, Faculdade de Ciências, Universidade de Lisboa, Lisboa, Portugal; Tianjin University, CHINA

## Abstract

Many biomedical relation extraction approaches are based on supervised machine learning, requiring an annotated corpus. Distant supervision aims at training a classifier by combining a knowledge base with a corpus, reducing the amount of manual effort necessary. This is particularly useful for biomedicine because many databases and ontologies have been made available for many biological processes, while the availability of annotated corpora is still limited. We studied the extraction of microRNA-gene relations from text. MicroRNA regulation is an important biological process due to its close association with human diseases. The proposed method, IBRel, is based on distantly supervised multi-instance learning. We evaluated IBRel on three datasets, and the results were compared with a co-occurrence approach as well as a supervised machine learning algorithm. While supervised learning outperformed on two of those datasets, IBRel obtained an F-score 28.3 percentage points higher on the dataset for which there was no training set developed specifically. To demonstrate the applicability of IBRel, we used it to extract 27 miRNA-gene relations from recently published papers about cystic fibrosis. Our results demonstrate that our method can be successfully used to extract relations from literature about a biological process without an annotated corpus. The source code and data used in this study are available at https://github.com/AndreLamurias/IBRel.

## Introduction

One of the major sources of current scientific knowledge is scientific literature, in the form of articles, patents and other types of written reports. This is still the standard method researchers use to share their findings. However, it is essential that a research group working on a certain topic is aware of the work that has been done on the same topic by other groups. This task requires manual effort and may take a long time to complete, due to the amount of published literature. One of the largest sources of biomedical literature is the MEDLINE database, created in 1965. This database contains over 26 million references to journal articles in life sciences, while more than 800,000 were added in 2015.

Automatic methods for Information Retrieval and Information Extraction aim at obtaining relevant information from large datasets, where manual methods would be infeasible. When applied to literature, this task is known as text mining. Named entity recognition is a text mining task that aims at identifying the segments of text that refer to an entity or term of interest. Another task is normalization, which consists of assigning an ontology concept identifier to each recognized entity. Finally, the relations described between the identified entities can be extracted, which is known as relation extraction. The language used for scientific communication is formal, but the names of the biomedical entities may not be consistent across different papers. Nonetheless, text mining has been applied successfully to biomedical documents, for example, to identify drugs [1] and protein-protein interactions [2]. Supervised machine learning can be used to train a relation classifier. This approach requires an annotated corpus so that the algorithm can learn to predict the label of new instances. The algorithms that have been used for this task are, for example, conditional random fields [3] and kernel methods [4], based on shallow linguistic information [5] and parse trees [6].

In some domains, such as microRNA regulation, there is a limited amount of annotated corpora to train systems due to the cost of manually annotating text. MicroRNAs, or miRNAs, are small endogenous sequences of nucleotides used by animals, plants, and viruses to downregulate gene expression by targeting messenger RNA for cleavage or translational repression [7]. Since they were discovered, these molecules have been found to be associated with several biological processes, including various developmental and physiological processes. For this reason, their dysfunction might contribute to human diseases [8, 9]. The expression of each miRNA is regulated by transcription factors. Therefore, these regulatory relations provide an interesting case study of complex biological processes, where miRNAs are regulated upstream by transcription factors, while miRNAs target specific genes, and each miRNA-gene pair may be associated with one or more diseases. miRNAs are considered potential diagnostic and therapeutic targets for complex diseases [10]. As of September 2016, a “miRNA” keyword search on PubMed retrieves 52144 citations, of which 39568 were published in the last 5 years. The knowledge contained in these documents is of great importance to researchers working on a specific disease since it could lead to the formulation of new hypothesis.

Several databases have been created to improve the quality of the current miRNA knowledge. One of these databases, miRBase, indexes the reference names, sequences, and annotations of newly discovered miRNAs [11]. This initiative is particularly important in order to keep the nomenclature of all miRNAs consistent and unambiguous.

The Human MicroRNA Disease Database stores associations between miRNAs and diseases supported experimentally [12]. Another database, miRTarBase [13], provides information about miRNA-target relations, based on experimental data published in papers. Furthermore, this database provides a user interface with several features, such as visualization of miRNA-target networks, and an error report system. The authors update this database regularly, using natural language processing tools to choose which papers should be integrated. The reduce the curators’ workload, the developers of this database added a text mining module on its latest release, contributing to an increase in the number of relations by 7-fold, comparing to the previous version. Chowdhary et al. [14] proposed a database for respiratory and related diseases, where the promoter regions of genes associated with these diseases are annotated with TFs and TF binding sites. With this database, it is also possible to compare genes, TFs, GO terms and miRNAs associated with selected diseases.

This increased interest in miRNA regulation has led to the development of computational methods to extract evidence based miRNA associations with genes, targets and diseases [15]. Computational methods provide various advantages over experimental methods, such as higher reproducibility and lower costs. The main techniques used to develop these methods are semantic similarity, network analysis and machine learning [16]. TFmiR is a web tool to analyze relations between miRNAs, transcription factors and genes of a specific disease, exploring the information contained in various knowledge bases [17]. This tool takes as input a list of miRNAs and genes and performs network analysis according to user input scenarios. The authors were able to identify core regulators and TF-miRNA regulatory motifs that were confirmed to be described in the literature. Liu et. al. [18] identified potential miRNA-disease relations by combining a disease network with a miRNA network based on miRNA-disease associations known from the Human MicroRNA Disease Database. Using miR-isomiRExp, it is possible to cluster miRNA isoforms according to their expression pattern [19]. This type of analysis can be advantageous to understand miRNA maturation, processing mechanisms, and functional characteristics.

Recently, text mining approaches have been used to extract information about miRNA regulation. Murray et al. [20] extracted miRNA-target relations from PubMed using a list of verbal phrases, chosen to extract regulatory and functional interactions. Their method identified (miRNA, verb, gene) triplets, which were then manually validated, to reduce the number of false positives. The authors were able to identify 1165 miRNA-gene relations, which they used to generate a network. By aggregating relations described in multiple papers, they obtained a snapshot of the miRNAome and linked miRNAs to biological processes and diseases based on their corresponding genes. However, they did not evaluate the extraction process against a gold standard, and hence we were not able to compare their results to other works in terms of precision and recall.

miRSel is a database of miRNA-gene relations which uses text mining methods to automatically update its entries [21]. The authors extracted miRNA entities using regular expressions and gene entities based on a dictionary compiled from several databases. Similarly to Murray et. al., they also compiled a list of 70 expressions used to describe miRNA-gene relations and extracted the instances where a miRNA, gene, and expression co-occurred. They evaluated their method on a set of 89 sentences from PubMed abstracts, obtaining an F-score of 0.83.

The developers of OMIT (Ontology for MicroRNA Target) explored automatic methods to find new miRNA terms to add to the ontology [22]. They obtained abstracts related to miRNA through keyword search on PubMed and filtered out the terms that were already mapped to the ontology. Then, nouns and noun phrases that did not match with existing ontology concepts were considered candidate terms. The most frequent candidate terms were then reviewed by domain experts and added to the ontology when appropriate. Starting with 49,447 abstracts and 488,576 nouns and noun phrases, the authors were able to add 117 new terms to the ontology. This type of approach can be enhanced by using a more advanced term extraction method, in order to present only high confidence candidates to the domain experts, requiring less manual effort.

Bagewadi et. al [23] compared various approaches to miRNA-gene relation extraction, including co-occurrence and machine learning algorithms. To evaluate these approaches, they manually annotated a corpus of 301 abstracts with various types of entities, including miRNAs and genes/proteins, and with the relations described in each sentence. Using the supervised machine learning approach, their best F-score was 0.76, while using a co-occurrence approach their best F-score was 0.73.

Li et. al [24] developed miRTex, which extracts miRNA-gene relations based on a set lexico-syntactic rules. They developed an annotated corpus of 150 abstracts to evaluate their system, which obtained the F-score of 0.94 for miRNA-gene relation extraction. Then, they applied their system to a set of 13M abstracts and 1M full-text documents and released a database containing those results. The authors obtained an F-score of 0.87 on Bagewadi et al.’s corpus. However, their method was based on hand-crafted rules and lists of keywords which are difficult to generalize and require costly manual curation. Although they obtained high F-score values for miRNA-gene relation extraction, it is not clear how their methods could be efficiently applied to other datasets. This is a common issue of rule-based and supervised learning approaches.

It is not feasible to develop an annotated corpus for every domain since it is a time-consuming process and the annotations are likely to be biased to that particular corpus. Consequentially, there has been an increasing interest in semi-supervised and unsupervised approaches to perform relation extraction. Fully unsupervised approaches explore clustering algorithms to identify patterns in the text that could indicate the presence of a relation. For example, Rosenfeld et al. [25] clustered pairs of entities using context features related both to the pair and to each entity, obtaining high precision levels. Alternatively, other authors have developed bootstrapping methods based on a small set of relations [26].

Distant supervision (sometimes referred to as weak supervision) combines advantages of both supervised and unsupervised learning [27]. This technique assumes that any sentence that mentions a pair of entities corresponding to a knowledge base entry is likely to describe a relation between those entities. For example, any sentence mentioning “Nikola Tesla” and “New York” would be identified as a positive example of a “lived in” relation. This would include sentences such as “Nikola Tesla lived in New York from 1933 to 1943” but also “Nikola Tesla planned the Wardenclyffe Tower facility in New York”, which does not in fact represent a “lived in” relation. However, the fact that a corpus of any size can be used as training data is an advantage over supervised learning, which is limited by the amount of documents manually annotated by experts. The pseudo-relations inferred using this technique can then be used to train a classifier using machine learning algorithms.

Multi-instance learning [28] addresses some limitations of distant supervision, by considering that not every co-occurrence will correspond to a relation mention. With this type of model, the pairs are grouped into bags where at least one of the pairs is true, but it is unknown if all pairs of the same bag are true. Riedel et al. [29] used this technique to extract Freebase relations from newspaper articles, obtaining a lower error rate than other distant supervision approaches. Min et al. [30] proposed an approach to reduce the number of incorrectly labeled relations, by considering only positive and unlabeled pairs. They found out that many of the pairs classified as negative from two distant supervision datasets were actually false negatives. These false negatives will have a significant impact on the performance of a classifier trained on those datasets.

Biomedicine is a challenging domain for text mining, due to the complexity of the studied processes. It is often necessary to train classifiers with a dataset annotated by domain experts with specific entities and relations due to the specialized terminology used to describe some processes. Distant supervision can overcome this necessity, by combining a set of documents with an existing knowledge base. These knowledge bases can be in the form of databases and ontologies, which already exist for many biological processes. Craven and Kumlien [31] have previously explored biomedical databases to generate training data for a relation extractor. They retrieved 924 abstracts that were referenced in the entries of the Yeast Protein Database and selected sentences that mentioned two entities corresponding to a database entry. Using these sentences, they trained a sentence classifier to extract subcellular locations of proteins. Their results demonstrated that weakly labeled data can be advantageous for relation extraction. Other authors have also explored this type of approach in the context of the biomedical domain [32, 33].

In this paper, we describe our method, IBRel—Identifying Biomedical Relations, which does not require a manually annotated corpus. To the best of our knowledge, this is the first biomedical relation extraction method based on multi-instance learning. Our method was based on the sparse multi-instance learning algorithm, used to train on an automatically generated corpus of 4,000 documents related to miRNAs. We evaluated IBRel on three datasets, comparing multi-instance learning with co-occurrence and supervised learning. IBRel was superior to supervised learning on one of three datasets, for which there was no specific training set available. To demonstrate how this method can be applied to a specific subject, we used it to extract relations from abstracts related to miRNA regulation and cystic fibrosis (CF). Recently, the role of miRNAs as therapeutic targets and in regulating cystic fibrosis transmembrane conductance regulator (CFTR) expression has been a topic of increasing interest to the CF research community [34, 35]. We were able to extract several miRNA-gene relations relevant to CF, highlighting how this work can lead to the improvement of our knowledge about human diseases.

## Materials and methods

### Corpora

Table 1 provides details about the corpora used for this work, both to develop (Dev) and evaluate (Eval) the system. Our objective was to perform a robust evaluation of our miRNA-gene relation extraction method. As such, the corpora used represented various annotations methodologies. TransmiR and miRTex were annotated only with document-level relations, while Bagewadi contained mention-level relations. Document-level relation annotations consist of a list of relations associated with each document, with no specific text span associated with each relation. When a corpus is annotated with mention-level relations, the location in the text of each annotated relation is known. The algorithms we used required mention-level relations for training, so both the miRTex and TransmiR corpus could not be used to develop the relation extraction system, but only for its evaluation. The IBRel-miRNA corpus contains more documents and entities than the others because it was automatically generated. The purpose of this corpus was to develop an approach based on distant supervision. We applied our method on a corpus of abstracts about cystic fibrosis and miRNAs, in order to demonstrate how IBRel can be used to obtain knowledge about a specific disease.

**Table 1:** Corpora used to develop and evaluate the system. Each line refers to a corpus, how it was used (Dev: development; Eval: evaluation; NER: Named Entity Recognition; RE: Relation extraction), the total number of relevant entities and relations annotated, and the number of documents.

	NER		RE				
Corpus	Dev	Eval	Dev	Eval	Entities	Relations	Documents
Bagewadi’s	X	X	X	X	1963	318	301
miRTex	X	X		X	1245	771	350
TransmiR		X		X	1145	547	243
IBRel-miRNA			X		52970	NA	4000
IBRel-CF				X	612	NA	51

We used the training set of Bagewadi’s corpus [23] to train a miRNA-gene relation classifier as well as classifiers for miRNA and gene entity recognition. Furthermore, we used the respective test set to evaluate miRNA and gene entity recognition and miRNA-gene mention-level relation extraction. Bagewadi’s corpus consisted of MEDLINE abstracts, selected using the keyword “miRNA”. The authors annotated 301 documents with specific and non-specific miRNAs, Gene/Proteins, Diseases, Species, and Relations Triggers, as well as undirected relations between entities mentioned in the same sentence. The inter-annotator agreement score was 0.916 for specific miRNAs and 0.752 for Gene/Proteins.

We used the miRTex corpus [24] to evaluate miRNA and gene entity recognition, as well as document-level miRNA-gene relation extraction. This corpus was annotated with miRNA and genes entities, and with three types of relations: miRNA-gene, miRNA-target, and gene-miRNA. A relation was classified as miRNA-target if a direct interaction between a miRNA and gene was described. In this corpus, the relations were annotated only at document-level, i.e. no specific text span associated with each relation. The inter-annotator agreement for the relations was 0.86, determined for a set of 20 abstracts.

TransmiR is a database that stores transcription factor-miRNA regulatory relationships found in the literature [36]. In this study, we created the TransmiR corpus, based on the document abstracts associated with the entries related to humans of this database (S1 Dataset). The abstracts were retrieved from PubMed using the identifiers provided with each entry. Since one of the fields of each entry of this database was “organism”, we used every entry that had something other than “human” as the knowledge base for distant supervision. There were three abstracts that were not available on PubMed (PMIDs 17972953, 20046097 and 18818704), resulting in a total of 243 abstracts. Each abstract was annotated with the miRNA-gene relations that exist in the database. Our objective was to determine if we can obtain the same relations using our method.

Distant supervision requires a large corpus and a knowledge base containing the type of relations to be extracted. Regarding the knowledge base, we used the non-human entries of the TransmiR database to avoid overlap with the TransmiR corpus. Furthermore, we obtained 4,000 documents about miRNAs from PubMed, using the MeSH term “miRNA”, ordered by publication date. We refer to this corpus as IBRel-miRNA corpus (S2 Dataset). This corpus consisted uniquely of these documents, without any type of annotation. However, to use it for distant supervision, we classified the text with named entity recognition classifiers, in order to obtain miRNA and gene named entities. This process is described in more detail in the “Biomedical Named Entity Recognition” section. Entities found were matched to the knowledge base to generate training data for the distant supervision model.

To demonstrate the usefulness of this technique to a particular real-world problem, we retrieved a corpus of 51 documents from PubMed, using the keywords “cystic fibrosis” and “miRNA” (IBRel-CF corpus). Similarly to the IBRel-miRNA corpus, we classified each document with named entity recognition classifiers, in order to obtain miRNA and gene named entities. Afterward, we classified each document with IBRel, as described in the “Identifying Biomedical Relations” section.

### Evaluation

**Fig 1:**
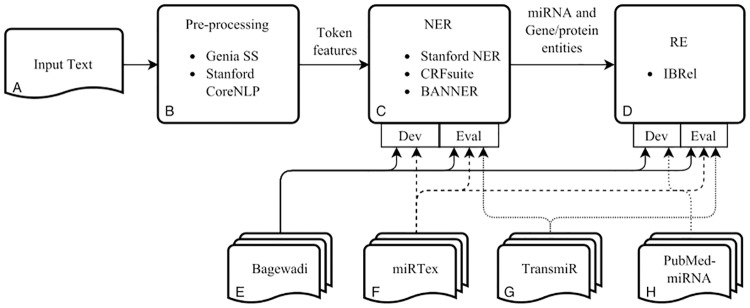
Pipeline used to perform the experiments. The input text (A) first goes through natural language processing tools to generate token features (B), then a named entity recognition module (C) to identify named entities and finally relation extraction (D) to extract relations between entities. Bagewadi (E), miRTex (F), TransmiR (G) and IBRel-miRNA (H) refer to the four corpora previously described.

Our experimental approach combined natural language processing techniques, as well as machine learning algorithms. The pipeline developed for this approach and the corpora used to evaluate each module are presented in Fig 1. The first module (B) processes the input text (A), extracting sentence and word boundaries, as well as token features such as lemma and part-of-speech. These features were necessary to develop and evaluate the other two modules. The NER module (C) consisted of named entity classifiers trained for miRNA and gene/protein entities, while the RE module (D) consisted of our method for miRNA-gene relation extraction, IBRel. Furthermore, we compared our method with supervised learning and co-occurrence approaches. Fig 1 also shows how each corpus was used, either to develop or evaluate the NER and RE modules. The corpora mentioned in Fig 1E-1H are the same ones mentioned in Table 1, except IBRel-CF, which was not used to develop or evaluate the system, but only as an independent case study.

As shown in Fig 1D, the miRNA-gene relation extraction module was evaluated on three corpora. Each corpus was developed using different methodologies and guidelines, therefore we consider this to be a robust evaluation. Using miRTex corpus, we studied the capacity to identify the relations of each document, while using Bagewadi’s corpus, we studied the capacity to identify each relation mention from the text. The TransmiR corpus evaluation provides a point of comparison to manually curated databases. However, this evaluation had some limitations. First, it is not possible to evaluate the extraction of relations independently from named entity recognition; if some entities are not correctly recognized, it will not be possible to extract relations that include those entities. Second, it may be the case that the system identified relations that were not in the database. However, it does not necessarily mean that they were incorrect since we used a different set of entries of the TransmiR database to train and evaluate the system: entries where the organism was different from “human” were used to train the model (IBRel-miRNA corpus) while the other entries were used for the TransmiR corpus gold standard. Third, we retrieved only abstracts related to the database entries. However, some relations from the database were not mentioned in the abstract, but only in the full text, figures, tables or supplementary material. These limitations should be taken into consideration when interpreting the results.

On Bagewadi’s corpus, which contained relation mentions, we considered a true positive if the offsets of the two entities of the pair matched the gold standard. For the other corpora, we normalized the text of each element of the relations to database identifiers from miRBase and UniProt [37]. This way, the possibility of false positives occurring due to nomenclature variation was reduced. We searched UniProt for the entry with the highest confidence that matched each protein entity, while for miRNAs we used a set of rules to match each miRNA entity to miRBase. We describe this process in greater detail in the “Biomedical Named Entity Recognition” section. Furthermore, we did not consider the direction of the relation when evaluating the results so that the order of the elements of each pair did not affect the results.

Three of the five corpora used were not annotated with named entities, hence it was necessary to perform and evaluate named entity recognition of miRNAs and genes. We used the test sets of miRTex and Bagewadi to evaluate this task since both were annotated with miRNA and gene mentions.

The evaluation measures used to evaluate the NER and RE modules were precision, recall, and F-score. These measures are commonly used to compare the performance of relation extraction methods on community challenges [2, 38]. Precision corresponds the fraction of relations retrieved that were relevant, while recall corresponds to the fraction of all relevant relations that were retrieved by the method. F-score corresponds to the harmonic mean between precision and recall. This measure it particularly important since it is usually trivial to obtain either high precision at the expense of a low recall, or vice-versa. However, these measures depend on the distribution of the corpus [39], so it can be difficult to compare results across different test sets.

### Identifying biomedical relations

Our objective was to identify miRNA-gene regulatory relations in scientific abstracts without requiring additional manual data curation. We present a method, IBRel, to extract biomedical relations based on distant supervision. Our method requires only a set of documents, which can be easily retrieved from PubMed, and a knowledge base, which already exists for many biological problems. We focused on miRNA regulatory relations and selected the TransmiR database as the knowledge base. Each miRNA-gene pair mentioned in the same sentence was considered a potential miRNA-gene relation mention. These relations could be either a miRNA regulating the activity of a gene, or a gene or protein regulating the transcription of a miRNA. Existing approaches to extract this type of relation are based on fixed rules, which are difficult to adapt to other relations, or manually annotated corpora, which are costly to produce. Therefore, we considered miRNA regulation a relevant case-study to demonstrate the usefulness of IBRel to biological problems given the lack of annotated corpora.

The proposed method required a corpus to generate training instances. This corpus had to be larger than any other miRNA-gene corpora in terms of number of documents, and it should contain entities and relations relevant to miRNA regulation, i.e. the text should contain instances of miRNA-gene relations. We retrieved a corpus of 4,000 abstracts from PubMed, using the MeSH term “miRNA” (IBRel-miRNA corpus). Firstly, we applied a NER algorithm to recognize the miRNA and gene entities in this corpus. The NER algorithm was based on a machine learning classifier trained on the Bagewadi and BioCreative 2 GM task datasets, and both classifiers were evaluated on gold standards. Using the IBRel-miRNA corpus and the recognized entities, we trained a classifier for miRNA-gene relation extraction.

To train IBRel, we used the sparse multi-instance learning algorithm (sMIL) [40]. The sMIL algorithm is based on the assumption that the bags are sparse, meaning that only a few instances are positive in each bag. Although this algorithm was first applied to image classification, other authors have used it for relation extraction [41]. An abstract may mention each miRNA and gene multiple times but generally, due to word restrictions, the relation will be stated only once. This is the reason why this variation of multi-instance learning was chosen for this task.

It was necessary to define how the sMIL algorithm would be integrated into our method to extract biomedical relations. Multi-instance learning differs from traditional supervised learning in the sense that instead of using a training set composed of labeled instances it uses a training set composed of labeled bags of instances. The main challenge in adapting multi-instance learning to the biomedical domain was defining how to represent the data in the form of bags. In our case, each bag contains multiple relations. These bags can be positive, if at least one of the instances corresponds to a true relation, or negative if no instances in that bag are true. In a biomedical abstract, a given miRNA and gene may co-occur several times, while only some of those instances correspond to the description of a miRNA-gene relation. Take into consideration the sentence: “These abnormalities reflect the regulation of a cadre of modulators of SRF activity and actin dynamics by miR-143 and miR-145.” (PMID 19720868); a relation is described between the gene SRF and two miRNAs. However, in the following sentence of that document: “Thus, miR-143 and miR-145 act as integral components of the regulatory network whereby SRF controls cytoskeletal remodeling and phenotypic switching of SMCs during vascular disease.”, the same miRNAs and gene are mentioned but no relation is described.

**Fig 2:**
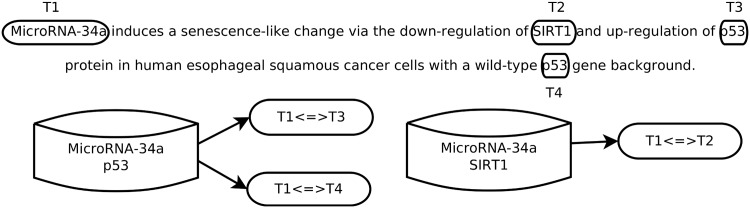
Multi-instance learning bags. For each sentence, we generated bags according to the distinct miRNA-gene pairs mentioned in the text. If a pair exists in the reference database, the bag is labeled as positive. Multi-instance learning assumes that at least one of the instances of a positive bag should describe a true relation.

To generate the bags for the sMIL algorithm, we considered an instance as a miRNA and gene co-occurrence in a sentence (Algorithm 1). Fig 2 contains an example of a sentence that produces one bag with two instances and another bag with one instance. The features used to represent each instance consisted of the words used before, between and after the two elements of the pair as well as their respective lemma, part-of-speech and named-entity tag (Person, Location, Organization, Numerical, Temporal, or Other) (Example 1). The size of the word window used was variable, and we experimented with window sizes 1, 3 and 5. We then converted these features into a bag-of-words representation using sci-kit learn [42]. These features were selected with the objective of being similar to the ones used by the supervised machine learning algorithm we chose to compare with IBRel.

**Algorithm 1** Bag generation algorithm

1: **function** GENERATE_BAGS(corpus, transmir_human)

2:  bags = []

3:  **for** sentence in corpus **do**

4:   **for** mirna in sentence **do**

5:    **for** gene in sentence **do**

6:     bag = (mirna, gene)

7:     instance_features = generate_features(bag, sentence)

8:     **if** bag not in bags **then**

9:      **if** bag in transmir_human **then**

10:       bag.label = 1

11:      **else**

12:       bag.label = 0

13:      bags.add(bag)

14:     bags.add_instance_to_bag(bag, instance_features, bag_label)

15:   **return** bags

**Example 1.** Sparse Multi-instance learning instance example.

- **Sentence:** These abnormalities reflect the regulation of a cadre of modulators of SRF activity and actin dynamics by miR-143 and miR-145. (PMID 19720868)
- **Pair:** miR-143—SRF
- **Label:** 1
- **Feature vector:** (0-before-cadre-NN-O 1-before-of-IN-O 2-before-modulators-NNS-O 3-before-of-IN-O 0-middle-activity-NN-O 1-middle-and-CC-O 2-middle-actin-NN-O 3-middle-dynamics-NNS-O 4-middle-by-IN-O 0-end-and-CC-O 1-end-miR+145-NN-O 2-end-.-.-O)

We did not manually annotate the relations of the training corpus, so it was necessary to explore a knowledge base to assign labels. This knowledge base had to contain relations of the same type as the ones to be extracted. For this purpose, we used the entries from TransmiR that were not related to the human species. This way, we avoided overlapping with the TransmiR corpus used for evaluation, which was generated using only the human entries. Each TransmiR entry contains a miRNA identifier as well as a gene name. We used these two columns to match with the miRNAs and genes found in the text. As shown in Algorithm 1, if the miRNA-gene pair existed in the human TransmiR database, the bag label was 1, otherwise, it was 0.

We trained a classifier for miRNA-gene relation extraction on bags generated from the IBRel-miRNA corpus and the TransmiR database, following Algorithm 1. The sMIL algorithm learned a classification model from the training data as described in “Corpora” and implemented by the miSVM package (https://github.com/garydoranjr/misvm). We used the default values of miSVM since we did not want to overfit the classifier to a particular dataset.

We evaluated IBRel on three datasets (Bagewadi, miRTex, and TransmiR). Those three datasets were chosen because two of them were manually annotated with miRNA-gene annotations while the other one was obtained using TransmiR database entries that were not used to train the classifier. We generated instances from each document and bags containing those instances, as previously described. If a bag was classified as positive, every instance in that bag was also classified as positive.

The confidence level of each prediction was estimated using the distance to the hyperplane, provided by the support vector machines algorithm. We used a logistic link function to obtain the probability output, as suggested by Wahba [43]. This probability was given by Eq (1), where *f*(*x*) is the uncalibrated value returned by the SVM.

$$\begin{array}{c} P\left(class|input\right)=P\left(y=1|x\right)=p\left(x\right)=\frac{1}{1+exp(-f(x))} \end{array}$$ (1)

If a relation was found in more than one document, we used the maximum confidence level obtained.

### Supervised machine learning and co-occurrence approaches

To assess the performance of IBRel on miRNA-gene relation extraction, we performed the same analysis using two other relation extraction approaches. First, we applied a co-occurrence approach which consisted in classifying every miRNA-gene pair in the same sentence as positive. This approach is considerably faster but tends to overestimate the number of relations, producing more false positives. However, some authors have obtained strong results using co-occurrence for relation extraction [23, 44]. For example, Bagewadi et. al. mention that their co-occurrence approach obtains similar results of machine learning approaches. The assumption is that due to restrictions on the word number in abstracts, a sentence that mentions two entities is likely to describe a relation between those two entities.

As another comparative approach, we used a variation of support vector machines, with a shallow linguistic kernel, as implemented by Giuliano et al. [5], to train a classifier on an annotated corpus. The advantage of kernel methods such as this one is the fact that no features have to be designed and tested. This kernel compares the sequence of tokens, lemmas, part-of-speech and named entities of each instance with the others. Tokens that refer to each argument are identified and substituted by a generic string so that the original text does not affect the algorithm. The label of each instance was 0 if it described relation, or 1 if it did not describe a relation. Example 2 provides a feature vector of a pair instance of this algorithm. Each element corresponds to a token and is constituted by its order in the sentence, original text, lemma, part-of-speech, named-entity tag (Person, Location, Organization, Numerical, Temporal, or Other) and candidate identifier (A—Agent, T—Target).

**Example 2.** Shallow Linguistic Kernel instance example.

- **Sentence:** These abnormalities reflect the regulation of a cadre of modulators of SRF activity and actin dynamics by miR-143 and miR-145. (PMID 19720868)
- **Pair:** miR-143—SRF
- **Label:** 1
- **Feature vector:** (0/These/these/DT/O/O, 1/abnormalities/abnormality/NNS/O/O, 2/reflect/reflect/VBP/O/O, 3/the/the/DT/O/O, 4/regulation/regulation/NN/O/O, 5/of/of/IN/O/O, 6/a/a/DT/O/O, 7/cadre/cadre/NN/O/O, 8/of/of/IN/O/O, 9/modulators/modulator/NNS/O/O, 10/of/of/IN/O/O, 11/#candidateb#/#candidateb#/NN/ENTITY/T, 12/activity/activity/NN/O/O, 13/and/and/CC/O/O, 14/actin/actin/NN/O/O, 15/dynamics/dynamics/NNS/O/O, 16/by/by/IN/O/O, 17/#candidatea#/#candidatea#/NN/ENTITY/A, 18/and/and/CC/O/O, 19/miR-145/mir-145/NN/ENTITY/O, 20/./././O/O)

This kernel has been applied to biomedical text, for the extraction of relations between proteins [5] and chemical compounds [45]. The shallow linguistic kernel is a composite sequence kernel that uses both a local and global context window. We performed experiments using windows with size 1, 3 and 5. We used Bagewadi’s corpus to train a miRNA-gene relation classifier using this kernel since this was the only corpus available that was manually annotated with that type of relation mention.

### Biomedical named entity recognition

The recognition of biomedical entities is a critical step to our method because the algorithms used require these entities to be annotated in the text. While gene/protein named entity recognition is a task for which many systems have been developed, the same is not true regarding miRNAs. It was necessary to develop a method to recognize miRNA entities and evaluate both gene and miRNA named entity recognition methods. Then, each entity recognized was mapped to a database identifier. This step improves the quality of the information extracted by reducing lexical variation and by integrating external domain knowledge.

We applied an existing system for gene/protein named entity recognition, BANNER [46]. This system was evaluated on the three test sets used since we could not find published results on those datasets. BANNER is based on the conditional random fields algorithm [47]. This is a state-of-the-art algorithm used by NER systems that learns the patterns of tokens from an annotated gold standard. The model generated is then able to classify new text according to those patterns. BANNER contains a specific set of features based on orthographic, morphological and shallow syntax features. We used the model they trained for protein and gene named entity recognition on the BioCreative 2 GM task dataset. BANNER assigned a label to each token, expressing if that token was part of an entity or not.

We used the UniProt API to obtain the entry names corresponding to each gene entity. Example 3 provides an example of the query used, as well as the output obtained. Since this API does not provide a confidence score, we selected only the first entry obtained when sorted by their own internal score. Entities that were not mapped to the reference database were excluded. Since we are working with published papers, it is unlikely that the genes and proteins mentioned would be missing from the databases. UniProt was chosen instead of a more gene specific database to match both protein and gene entities to database identifiers because we wanted to identify as many entities as possible. Table 2 provides various examples of genes and proteins mapped to UniProt.

**Table 2:** Example of gene entities identified that were then matched with UniProt entries. Entity text refers to the original text found in the abstract, while Entry name and Entry ID refer to UniProt entries.

Entity text	Entry name	Entry ID
Smad	SMAD3_HUMAN	P84022
N-Myc	NDRG1_HUMAN	Q92597
Interferon regulatory factor 3	IRF3_HUMAN	Q14653
Egr-2	EGR2_HUMAN	P11161

**Example 3.** UniProt API example query and its output http://www.uniprot.org/uniprot/?query=insulin&sort=score&columns=id,entry%20name,reviewed,protein%20names,organism,&format=tab&limit=1

Output: P06213 INSR_HUMAN reviewed Insulin receptor (IR) (EC 2.7.10.1) (CD antigen CD220) [Cleaved into: Insulin receptor subunit alpha; Insulin receptor subunit beta] Homo sapiens (Human)

We performed basic pre-processing on the input text to extract features to train miRNA named entity classifiers on the text. Our system first splits the text into sentences, using the GENIA sentence splitter [48]. Each sentence is then processed by Stanford CoreNLP pipeline [49], to tokenize and extract lemmas, part-of-speech tags and named entity tags (proper noun, numerical or temporal entities) from the text.

We trained conditional random field classifiers on Bagewadi’s corpus for miRNA named entity recognition. For each corpus, we trained two classifiers: one using Stanford NER with the default features and another with CRFsuite, using the features described in [50]. Our objective was to maximize the number and variety of entities found since this is a limiting step for relation extraction. It has been shown that combining classifiers training with different implementations and features can improve the performance of a text mining system [51].

miRNA entities were mapped to a list of human miRNA names extracted from miRBase, which includes the names of mature and pre-mature miRNAs, as well as deprecated names. We used some rules to reduce the variation of miRNA entities, in order to obtain better miRBase matches. These rules were based on the most common spelling variations of miRNAs. Sometimes authors mention multiple miRNA at the same time, for example: “mir-192/215”, “mir-34a/b/c”, “mir-143 and -145”. We split a miRNA entity if it contained “/”, “and” or “,”. However, this rule was not applied to Bagewadi’s corpus because the guidelines specified that multiple miRNAs mentioned sequentially should be annotated as only one. Although miRNA nomenclature is well defined, some slight deviations appear in the literature. For example, sometimes “microRNA-” and “miRNA-” is used instead of “miR-”. In some papers, there is no dash connecting the “miR-” prefix to the respective number, for example, “miR125a”. Furthermore, human miRBase entries contain a “hsa-” prefix, which is not always used in the literature. We used simple post-processing rules to fix these variations. Then, each entity was matched to the list of miRNAs from miRBase, using fuzzy string matching. The confidence score of each match corresponded to the Levenshtein distance between the original text and the match. The Levenshtein distance is a string metric which is related to the minimum number of edits necessary to transform one string into another. Based on our experiments, we ignored matches with scores lower than 0.95 since many matches with those scores were incorrect. Table 3 provides some examples of the normalization process for miRNAs.

**Table 3:** Example of miRNA entities identified that were then matched with miRBase entries. Entity text refers to the original text found in the abstract, while Entry name and Entry ID refer to miRBase entries.

Entity text	Entry name	Entry ID
miRNA-155	hsa-miR-155	MI0000681
miR-200	hsa-miR-200a	MI0000737
miR125a	hsa-mir-125a	MI0000469
microRNA-9	hsa-mir-9	MI0000466

## Results

We evaluated miRNA-gene relation extraction on three datasets: Bagewadi, miRTex and TransmiR (Fig 1D-1H). These were the datasets which were annotated with miRNA-gene relations, although TransmiR was annotated automatically. Table 4 presents the miRNA-gene relation extraction results on those datasets. The co-occurrence approach consisted in classifying as true every miRNA-gene pair co-occurring in the same sentence We evaluated supervised learning with a shallow linguistic kernel (SL kernel), using a classifier trained on Bagewadi’s corpus (supervised learning) and our method, IBRel, using a classifier trained on the IBRel-miRNA corpus. We used a fixed window of 3 on the SL kernel and IBRel, while we provide results for windows of size 1 and 5 in S4 Dataset.

**Table 4:** miRNA-gene relations extraction evaluation results on each corpus, comparing co-occurrence, supervised and IBRel (window size = 3). P, R and F refer to precision, recall and F-score.

	Co-occurrence			SL kernel			IBRel		
Gold standard	P	R	F	P	R	F	P	R	F
Bagewadi’s	0.528	0.992	0.689	0.661	0.886	**0.757**	0.493	0.577	0.532
miRTex	0.474	0.910	0.623	0.536	0.837	**0.654**	0.583	0.285	0.383
TransmiR	0.147	0.851	0.250	0.238	0.090	0.130	0.359	0.486	**0.413**

Comparing the three methods in terms of F-score, the shallow linguistic kernel approach obtains the best score on two corpora (Bagewadi and miRTex), while the IBRel outperformed the others on the TransmiR corpus. Comparing in terms of precision, IBRel obtained the best score on two corpora (miRTex and Bagewadi), while the shallow linguistic kernel obtained the highest score on Bagewadi’s corpus. With all three methods, the highest F-score obtained was on Bagewadi’s corpus. However, the F-score obtained for miRTex and TransmiR using IBRel was close (0.413 and 0.383), while for the other two approaches, the F-score on TransmiR is lower than on miRTex (co-occurrence: 0.623 and 0.25; kernel: 0.654 and 0.130).

We also evaluated miRNA and gene entity recognition using conditional random fields on the same datasets (Fig 1C and 1E-1H) since this is a required step for the relation extraction approaches we used (Table 5). For Bagewadi and miRTex, we used the respective training and test sets to recognize miRNA entities, merging the results obtained with two conditional random fields classifiers. For the TransmiR corpus, we used the classifiers trained on the miRTex corpus, which obtained higher values on its own evaluation. Gene entity recognition on every corpus was performed using BANNER. On TransmiR, the results obtained were lower than on the other two corpora, particularly for gene entities. This issue is related to how that corpus was developed and will be discussed in the following section.

**Table 5:** Entity recognition evaluation results on each corpus, for miRNA and gene entities. P, R and F refer to precision, recall and F-score.

	miRNA			Gene		
Gold standard	P	R	F	P	R	F
Bagewadi’s	0.902	0.936	0.919	0.814	0.580	0.677
miRTex	0.934	0.948	0.941	0.803	0.788	0.795
TransmiR	0.726	0.651	0.687	0.255	0.618	0.361

After evaluating our method, we used it extract miRNA-gene relations from a set of abstracts about cystic fibrosis and miRNAs (Table 6). The purpose of this study was to demonstrate the applicability of our method to a specific domain. These abstracts were removed from the training set (IBRel-miRNA corpus) to avoid any bias when developing IBRel Table 1. We were able to extract 27 relations, between 18 different miRNAs and 12 different genes. A total of 11 relations between the CFTR gene and a miRNA were found, which was to be expected since CFTR is the gene responsible for cystic fibrosis and the abstracts chosen dealt in most part with miRNA involvement in this disease. The maximum confidence level corresponds to the highest confidence of all instances of that particular relation. The confidence level of each instance was calculated by estimating the distance to the hyperplane, given by Eq (1). The relations with the highest confidence were also found in more sentences and abstracts.

**Table 6:** miRNA-gene relations extracted from the IBRel-CF corpus using IBRel, ordered by maximum confidence level.

miRNA	Gene	Sentences	Documents	Max. Confidence	Correct
hsa-mir-494	CFTR	10	5	0.996	Y
hsa-mir-93	CXCL8	6	1	0.978	Y
hsa-mir-101-1	CFTR	8	3	0.96	Y
hsa-mir-224	SLC4A4	5	1	0.937	Y
hsa-mir-145	CFTR	5	3	0.871	Y
hsa-mir-193b	BRCA1	2	1	0.86	N
hsa-mir-193b	CFTR	2	1	0.857	Y
hsa-mir-155	AKT1	4	1	0.828	Y
hsa-miR-199a-5p	AKT1	5	1	0.807	Y
hsa-mir-183	IDH2	3	1	0.763	Y
hsa-mir-155	CXCL8	5	2	0.736	Y
hsa-mir-125b-1	CFTR	4	1	0.709	Y
hsa-mir-125a	LIN28A	5	1	0.705	N
hsa-mir-224	CFTR	4	1	0.655	Y
hsa-mir-99b	LIN28A	5	1	0.651	N
hsa-mir-99b	KRT18	3	1	0.65	N
hsa-mir-126	TOM1L1	2	1	0.647	Y
hsa-miR-199a-5p	CAV1	5	1	0.642	Y
hsa-miR-509-3p	CFTR	3	2	0.613	Y
hsa-mir-125a	KRT18	3	1	0.58	N
hsa-mir-221	ATF6	3	1	0.546	Y
hsa-mir-145	SMAD3	3	1	0.543	Y
hsa-mir-138-1	CFTR	3	1	0.539	Y
hsa-mir-99b	CFTR	2	1	0.519	Y
hsa-mir-223	CFTR	3	1	0.513	Y
hsa-mir-125a	CFTR	2	1	0.512	Y
hsa-let-7e	LIN28A	3	1	0.508	N

## Discussion

Our method obtained better results when applied to the TransmiR corpus. When compared to the supervised learning approach, the F-score on this corpus was improved by 0.283 with our method. For example, the supervised classifier was not able to identify the miRNA-gene relations in “Hence, miR-192 and miR-215 can act as effectors as well as regulators of p53” (PMID 19074875), while IBRel identified both relations. Consequentially, we were not able to find any relations described similarly to that example in Bagewadi’s corpus. This type of error contributed to the difference in recall. Using a larger corpus, more sentence structures are taken into account, leading to a more flexible classifier.

On the miRTex corpus, our method obtained higher precision but lower recall, resulting in a lower F-score (0.383). It was not possible to train a classifier on this corpus using supervised learning since it was not annotated with relation mentions. For this reason, we used the classifier trained on Bagewadi’s corpus. The increase in precision of 0.047 using distant supervision on miRTex corpus reinforces the idea that our approach is more adaptable.

The supervised learning approach obtained higher results on the Bagewadi and miRTex corpora. Since the training set was annotated with the same criteria as the test set, any classifier trained on that training set is more in the line with the test set annotations. The main source of error with the supervised learning approach were sentences where the miRNAs and genes had similar functions. For example, in the sentence “These data implicate hsa-miR-30b, hsa-miR-30d and KHDRBS3 as putative oncogenic target(s) of a novel recurrent medulloblastoma amplicon at 8q24.22-q24.23.” (PMID 19584924), there is no miRNA-gene relation, although the words used are similar to the ones that would be used if the relation was between a miRNA and gene.

The co-occurrence approach obtained the highest recall because it classified every miRNA-gene pair in a sentence as a true relation. The precision obtained for Bagewadi and miRTex was close to the other two approaches. This may be due to the fact that since they were manually annotated, the documents were more relevant for the type of relations annotated. The abstracts selected for those two corpora are more likely to contain sentences describing relations than a random selection. Therefore, miRNA-gene pairs in the same sentence would often be related. Compared to IBRel, the co-occurrence approach obtained better F-score on Bagewadi and miRTex. For the TransmiR corpus, our method outperformed co-occurrence on precision and F-score by 0.212 and 0.163, respectively. The TransmiR corpus has fewer relations per entity than Bagewadi and miRTex (Table 1), which may explain why our method performed better than co-occurrence in this case. Our method improved the results of the corpus where the co-occurrence approach was less effective.

Comparing our results to other published results on miRNA-gene relation extraction, the proposed method obtained lower F-score values. For example, Bagewadi et. al. [23] reported an F-score of 0.760 on their corpus. The authors used a linear kernel to obtain that result while using the SL kernel we obtain a similar F-score of 0.757. Using IBRel we obtained a lower F-score 0.532. However, these authors developed and evaluated their approach only on their dataset, which is understandable since they were the first to develop a manually annotated corpus containing information about miRNA-gene relations. Li et. al. [24] developed a rule-based approach to extract document-level relations, obtaining an F-score of 0.94 on their own manually annotated dataset (miRTex corpus) and 0.87 on Bagewadi’s corpus. In this case, our best F-score on their dataset was 0.654 using SL kernel and 0.383 using IBRel, which is lower than the values reported by the authors. However, the approach used by these authors cannot be easily adaptable to other domains. This is the reason why in relation extraction community challenges, teams generally use machine learning approaches instead of designing rules specific for that challenge. Since IBRel could be applied to any biomedical relation represented in a knowledge base, it has more reusability than rule-based methods, which are specific for a biological problem.

Since we did not annotate the IBRel-CF corpus, we manually evaluated the results obtained. We identified some relations extracted from the corpus that were not correct. From the 27 relations extracted, we identified 6 errors. There is no mention of the gene BRCA1 in the document where the relation between that gene and hsa-mir-193b was extracted. This was due to a mapping error, where the string “uPA”, referring to urokinase plasminogen activator (PLAU), was incorrectly mapped to BRCA1. This error could be fixed using acronym extension so that the extended form of the gene is mapped instead of the acronym. The three relations with the gene LIN28A are incorrect. Although this gene regulates the expression of several miRNAs, those relations were not described in the text. This error occurred because some miRNAs were recognized as genes, and in this case, they were incorrectly mapped to the LIN28A gene. One possible solution to this problem is to use semantic similarity to improve the mapping process. Considering that entities mentioned in the same sentence should be semantically related, PLAU would be more semantically similar to the other genes mentioned than BRCA1. Therefore, semantic similarity could be used as a threshold to choose better mappings.

### Evaluation of miRNA and gene entity recognition

We were able to recognize miRNA and gene entities from the three corpora. Regarding miRNAs, this task was not difficult since miRNA nomenclature is standardized and thus not as ambiguous as other biomedical entities. In the case of Bagewadi’s corpus, the F-score obtained was similar to the reported inter-annotator agreement (difference of 0.002 for miRNA and 0.075 for gene). On the miRTex corpus, we obtained higher F-score values for both miRNA and gene entities. The results obtained with the TransmiR corpus were lower since this evaluation was limited by some factors. The main one was the fact that not all relations were mentioned in the abstract of the articles. For example, every document with a relation between hsa-let-7a-1 and a gene also contained a relation between other miRNAs from the let-7 family and that gene. However, this was never mentioned in the abstract. This error accounted for 42 false negatives. Another type of error was due to some miRNAs and genes mentioned in the abstract that were not part of the TransmiR database. For example, PMID 20093556 mentions 6 miRNAs, but only one miRNA-gene relation exists in the database. This type of error contributed to lower precision values for miRNA and gene entity recognition when compared to the other two corpora.

## Conclusion

In this paper, we showed that our method performed better on a dataset based on a manually curated database, while, as expected, supervised learning performed better on manually annotated datasets, developed for text mining applications. The main contributions of this paper are IBRel, a method for extraction of biomedical relations from texts using only existing resources, and a dataset of miRNA-gene relations automatically extracted and manually validated. The method we developed was evaluated for miRNA-gene relation extraction, where it outperformed supervised learning on the case where no specific training set was available.

A second contribution is the dataset obtained using our method for cystic fibrosis. We applied IBRel to a set of 51 abstracts about cystic fibrosis, published in the last 5 years. From the 27 miRNA-gene relations extracted, 21 of those were found to be correct in the context of cystic fibrosis. While this approach was not flawless, it should be of interest to researchers working on this subject since there are still few reliable resources for identifying miRNA-gene relations in disease-specific contexts. We intend to apply this approach to other diseases and develop a platform to visualize the information extracted.

The results obtained in this work suggest that our method can still be improved. For example, we can optimize the parameters of miSVM to this task using cross-validation on the datasets used. We intend to use ontologies to better annotate the corpus generated for distant supervision. Semantic similarity has been used before to extract protein-protein interactions [52] and drug-target interactions [53]. By computing the semantic similarity between the entities mentioned in a document, we can identify which are more likely to be associated. The similarity between two genes can be calculated using the semantic similarity between the two sets of Gene Ontology terms annotated to them. We have previously explored semantic similarity techniques for drug name recognition [54] and drug-drug relation extraction [50]. We used semantic similarity between two chemical entities on ChEBI as a feature for an ensemble classifier, obtaining higher precision values. Another type of approach we wish to explore is crowd-sourcing. Other authors have used crowd-sourcing to improve multi-instance learning results [55]. The idea is to use machine learning algorithms to correctly classify a wide range of cases and use crowd-sourcing to solve the most difficult cases.

## Supporting information

S1 Dataset TransmiR corpus. Corpus generated using the abstracts referenced in the entries of the TransmiR database.
(TSV)

S2 Dataset IBRel-miRNA corpus. Corpus generated using the MeSH term “miRNA” and annotated automatically with miRNA and gene entities.
(TSV)

S3 Dataset IBRel-CF corpus. Corpus generated using the keywords “cystic fibrosis” and “miRNA” and annotated automatically with miRNA and gene entities.
(TSV)

S4 Dataset Results with variable window sizes. Results using SL kernel and IBRel with the window size parameter 1 and 5.
(ODS)
